# Clinical decisions surrounding genomic and proteomic testing among United States veterans treated for lung cancer within the Veterans Health Administration

**DOI:** 10.1186/s12911-017-0475-8

**Published:** 2017-05-30

**Authors:** Olga Efimova, Brygida Berse, Daniel W. Denhalter, Scott L. DuVall, Kelly K. Filipski, Michael Icardi, Michael J. Kelley, Julie A. Lynch

**Affiliations:** 1 Department of Veterans Affairs Salt Lake City Health Care System, 500 Foothill Drive, Salt Lake City, UT 84148 USA; 2 Boston University School of Medicine, 715 Albany Street, Boston, MA 02118 USA; 3 Veterans Healthcare Administration Bedford, 200 Springs Rd, Bedford, MA 01730 USA; 4 RTI International, 307 Waverley Oaks Rd, Waltham, MA 02452 USA; 5 University of Utah, 30 2000 E, Salt Lake City, UT 84112 USA; 6 National Cancer Institute, NIH, 9609 Medical Center Dr, Rockville, MD 20850 USA; 7 University of Iowa Carver College of Medicine, 200 Hawkins Drive, Iowa City, IA 52242 USA; 8 Iowa City VA Medical Center, 601 Highway 6 West, Iowa City, IA 52246-2208 USA; 9 Durham VA Medical Center, 508 Fulton St, Durham, NC 27705 USA; 10 Duke University School of Medicine, 2301 Erwin Rd, Durham, NC 27710 USA; 11 University of Massachusetts College of Nursing & Health Sciences, 100 Morrissey Blvd, Boston, MA 02125 USA

**Keywords:** Biomarker, Proteomic, Genomic, Testing algorithm, Non-small cell lung cancer, VeriStrat, Epidermal growth factor receptor, Tyrosine kinase inhibitor, Erlotinib, Clinical decision support

## Abstract

**Background:**

Current clinical guidelines recommend epidermal growth factor receptor (EGFR) mutational testing in patients with metastatic non-small cell lung cancer (NSCLC) to predict the benefit of the tyrosine kinase inhibitor erlotinib as first-line treatment. Proteomic (VeriStrat) testing is recommended for patients with EGFR negative or unknown status when erlotinib is being considered. Departure from this clinical algorithm can increase costs and may result in worse outcomes. We examined EGFR and proteomic testing among patients with NSCLC within the Department of Veterans Affairs (VA). We explored adherence to guidelines and the impact of test results on treatment decisions and cost of care.

**Methods:**

Proteomic and EGFR test results from 2013 to 2015 were merged with VA electronic health records and pharmacy data. Chart reviews were conducted. Cases were categorized based on the appropriateness of testing and treatment.

**Results:**

Of the 69 patients with NSCLC who underwent proteomic testing, 33 (48%) were EGFR-negative and 36 (52%) did not have documented EGFR status. We analyzed 138 clinical decisions surrounding EGFR/proteomic testing and erlotinib treatment. Most decisions (105, or 76%) were concordant with clinical practice guidelines. However, for 24 (17%) decisions documentation of testing or justification of treatment was inadequate, and 9 (7%) decisions represented clear departures from guidelines.

**Conclusion:**

EGFR testing, the least expensive clinical intervention analyzed in this study, was significantly underutilized or undocumented. The records of more than half of the patients lacked information on EGFR status. Our analysis illustrated several clinical scenarios where the timing of proteomic testing and erlotinib diverged from the recommended algorithm, resulting in excessive costs of care with no documented improvements in health outcomes.

## Background

Over the last 5 years, there has been a rapid increase in the number of biological markers included in clinical practice guidelines and in Food and Drug Administration (FDA) approvals for new treatments, particularly in cancer care. Methods for identifying biomarkers have also expanded. Transcriptomics, proteomics, and metabolomics are used to analyze a variety of patient specimens to characterize an individual or tumor biology, in an effort to assess risk of disease, diagnosis disease, refine prognosis, predict response to treatment, and monitor response to therapy and surveillance for disease recurrence. The White House recognized the importance of precision medicine and announced a $215 million investment to accelerate translation of genetic discoveries into individualized treatments. Yet, we know very little about how existing precision medicine tools are integrated into clinical care. Are precision medicine applications being implemented concordant with guidelines? How are test results impacting treatment decisions? Do biomarker tests and targeted treatments improve long term health outcomes and decrease costs of inappropriate treatment?

Lung cancer is the leading cause of cancer deaths worldwide accounting for about 27% of all cancer deaths. In 2016, 224,390 new cases and 158,080 deaths from lung cancer were expected in the United States [1]. Lung cancer disproportionally affects veterans, who experience a higher incidence than the general population [2]. The VA Central Cancer Registry (VACCR) shows that over the past decade, approximately 7600 veterans with lung cancer have been treated within the VA each year. The prevalence of smoking among veterans translates into a higher risk for lung cancer and lower rates of targetable mutations.

Although the 5-year survival of patients with metastatic non-small-cell lung cancer (NSCLC) is only about 1% [3], a greater understanding of lung cancer molecular biology has contributed to the development of several promising biomarkers that are companion diagnostics to targeted treatments. These include epidermal growth factor receptor (EGFR), anaplastic lymphoma kinase (ALK), receptor tyrosine kinase ROS1, and the programmed death-ligand 1 (PD-L1), recently approved by the FDA as a companion diagnostic to the monoclonal antibody pembrolizumab [4]. Patients with specific EGFR deletions and substitutions benefit from targeted treatment with EGFR-tyrosine kinase inhibitors (EGFR-TKIs). In 2013, FDA approved EGFR-TKI erlotinib (Tarceva) for first-line treatment of metastatic NSCLC patients whose tumors have EGFR exon 19 deletions or exon 21 (L858R) substitution mutations as detected by an FDA-approved test [5]. Thus, EGFR mutation testing has become crucial for the therapy algorithm in NSCLC recurrence or metastasis. EGFR testing in NSCLC was recommended for all newly diagnosed patients with advanced NSCLC of all histological subtypes, except for squamous cell carcinoma [6, 7]. EGFR mutations are rare in squamous NSCLC [6, 7], making it a separate disease from both histologic and genetic perspectives.

Guidelines for erlotinib use and EGFR testing have evolved gradually over the last decade. The current standard of care calls for EGFR sequencing in all advanced-stage (stage IV and recurrence) patients with NSCLC of all histological subtypes, except for squamous cell carcinoma [7, 8]. For patients who test positive for EGFR mutations, erlotinib is recommended as first-line therapy. Some also recommend testing of early-stage patients with non-squamous histology (in order to have the result ready in case of progression) and in never-smokers with any histology.

In addition to individuals with identifiable oncogenic EGFR mutations, other groups of NSCLC patients may benefit from targeted anti-EGFR therapy. Several molecular diagnostic tests help identify some of these patients [9]. The most widely used of those is a serum-based assay marketed as VeriStrat (Biodesix, Inc., Boulder, CO). It uses mass spectrometry to detect eight inflammatory proteins that correlate with survival outcomes in advanced NSCLC patients. In addition to prognostic information, VeriStrat (proteomic test) predicts benefit from anti-EGFR TKI treatment. However, this test is not considered a replacement for an EGFR mutation test. Rather, it is designed for NSCLC patients of any histology with a negative (wild-type) or unknown EGFR mutation status, who have progressed after or are ineligible for platinum-based chemotherapy. The blood-based test is particularly useful for patients for whom the EGFR test cannot be performed, for example due to an insufficient amount of biopsy material, or clinical indications against biopsy.

The proteomic test results are classified as “VeriStrat Good” (Good) or “VeriStrat Poor” (Poor). The “Poor” result indicates that the patient has poor prognosis with more aggressive disease and will benefit more from platinum-based chemotherapy than from EGFR-TKIs [10]. A meta-analysis of clinical data demonstrated that proteomic “Good” status predicted a better clinical outcome with a pooled hazard ratio of 0.40 (95% CI 0.32 to 0.49; p <0.001) for overall survival, and 0.49 (95% CI 0.39 to 0.60; p <0.001) for progression-free survival [11]. In patients with “Good” status, EGFR-TKI therapies and chemotherapy have the same survival outcome. The prognostic ability of the proteomic test has not been widely recognized; however, the test has been proven to be helpful for selecting and monitoring patients for EGFR-TKI treatment [9].

In this study, we analyzed use of this proteomic test within the VA between 2013 and 2015. We set out to determine whether testing was consistent with the intended use and current clinical practice guidelines. We also considered whether test results informed treatment decisions and cost of care.

## Methods

We conducted a retrospective cohort study using secondary data analysis methods. The primary sources of data were: the VA’s electronic medical records; Computerized Patient Record System; the VA’s Corporate Data Warehouse (CDW); VA pharmacy data, and patient-level proteomic test orders obtained from Biodesix (Boulder, Colorado), the laboratory which developed and conducts the test for VA medical centers (VAMCs). Proteomic test results were merged with structured electronic health record (EHR) data from the CDW. We used the pharmacy data to identify the start date, frequency and duration of erlotinib prescriptions.

Our cohort consisted of all patients (69) who underwent VeriStrat proteomic testing at the VA from August 2013 until February 2015. We conducted descriptive analyses to characterize these patients, and conducted detailed chart reviews on their medical records. Medical records were annotated by three annotators using ChartReview application developed by the VINCI development team [12]. The purpose of the chart review was to validate data reported from CDW, identify tumor histology, determine whether patients underwent a lung biopsy, capture the date of the lung biopsy, and determine whether EGFR mutational analysis was ordered. We calculated the time between EGFR testing, proteomic testing, and the first order of erlotinib treatment for each patient.

**Fig. 1:**
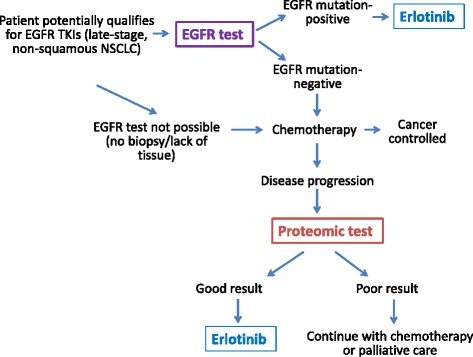
Algorithm for intended use of EGFR and proteomic testing in NSCLC patients. For clarity, various algorithms of chemotherapy and targeted treatments other than erlotinib were not included

We reviewed the clinical practice guidelines on EGFR testing issued by the National Comprehensive Cancer Network, the College of American Pathologists, the International Association for the Study of Lung Cancer, the Association for Molecular Pathology, and the American Society of Clinical Oncology [6, 7]. Based on those guidelines and the recommendations from Biodesix, we outlined a simplified clinical algorithm for EGFR and proteomic testing to predict erlotinib benefit (Fig. 1). This algorithm is intentionally simplified, focusing on erlotinib as the most established targeted therapy for metastatic NSCLC, and not on other targeted treatments, such as crizotinib against ALK1 and ROS1. We applied this algorithm to the collected patient data to determine whether EGFR testing and erlotinib treatment was concordant with the clinical guidelines, and whether proteomic testing was applied according to its intended use. We also analyzed the demographics and site of care, i.e., Veteran Integrated Service Network (VISNs) of patients tested.

We obtained permission to conduct this research from the VA Institutional Review Board (IRB) and the Research and Development Committee. The IRB authorized a waiver of both informed consent and Health Insurance Portability and Accountability Act authorization.

## Results

During the time of the study, 69 patients diagnosed with NSCLC underwent proteomic testing within the VA. The demographic characteristics of the patients tested are presented in Table 1.

**Table 1:** Characteristics of patients who underwent proteomic testing

Characteristics	Number	Percent
Age, Mean (SD)	69.2 (8.5)	
Age group		
50–59	7	10
60–69	31	44
70–79	24	34
≥ 80	7	10
Race/ethnicity		
Black or African American	12	19
White	48	76
Other	9	13
Vital status		
Alive	59	86
Deceased	10	14
Period of services		
Pre-Vietnam	18	26
Vietnam	43	62
Post-Vietnam	8	12
Agent orange exposure	60	87
Service connected		
100%	14	20
10 to 90%	15	22
Not service connected	40	58
Means status		
Exempt from copay	23	33
Discretionary	11	16
Not applicable or missing	35	51
Veteran Integrated Service Network		
VISN 1	10	15
VISN 7	12	17
VISN 17	13	19
VISN 20	12	17
7 Other VISNs	22	32

Analysis of VA administrative data as August 2015

Service connected represents percentage of care that is covered by the VA due resulting from a service-related injury

Means status indicates whether the Veteran is eligible for free or reduced cost of care

The age of patients tested ranged from 50 to 89 years (mean = 69.2, SD = 8.5). The majority of patients (48, or 76%) were White, 12 (19%) were Black or African American, and 9 patients were either of another race or declined to answer. Patients tested were treated in 11 out of existing 21 VISNs, with 68% of testing ordered by just 4 VISNs. The highest volume of testing was in VISN 17 (VA Heart of Texas Health Care Network) with 13 tests ordered, followed by VISN 7 (VA Southeast Network) and VISN 20 (Northwest Network), each with 12 tests, and VISN 1 (VA New England Healthcare System) with 10 tests. Out of the 69 patients tested, 39 were diagnosed with adenocarcinoma, 20 with squamous cell carcinoma, one with mixed type (squamous and adeno), one with large cell neuroendocrine carcinoma, and for the remaining cases tumor histology was not specified. Ten patients were deceased by the time of our analysis, 9 of whom died within three months of the proteomic test.

**Fig. 2:**
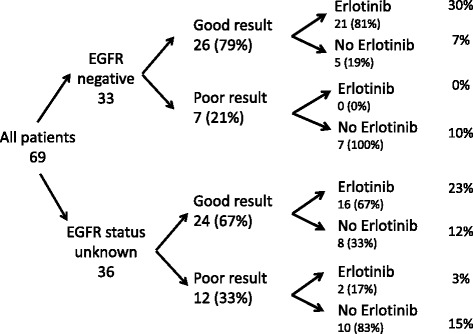
EGFR and proteomic test results and use of erlotinib. This diagram does not include the timing of testing and drug prescription

We analyzed test results, pharmacy data, and performed clinical chart reviews for the 69 patients in order to evaluate the concordance of EGFR and proteomic testing and erlotinib administration with recommended use. Proteomic testing should be administered only after the EGFR test has revealed no mutations, or if the EGFR mutational status is unknown, for example due to an insufficient amount of biopsy tissue. Because the proteomic test is serum-based, its use is not limited by tissue availability. We found that none of the 69 patients who received the proteomic test in our study had a documented EGFR driver mutation. Of the 69 patients, 33 (48%) tested negative for EGFR activating mutations, and for 36 (52%) EGFR status was not determined (Fig. 2), due to squamous histology, lack of tissue, or for unspecified reasons. Proteomic results were “Good” for 50 out of 69 patients (72%) and “Poor” for the remaining 19 patients (28%). Overall, of the 50 patients for whom the proteomic test revealed “Good” status, only 37 (74%) received erlotinib prescription, while 13 patients (26% of those with “Good” status, or 19% of the whole cohort) did not receive the drug. We also identified 2 patients, who received erlotinib despite their “Poor” status. Thus, the proteomic test and subsequent erlotinib treatment were used according to our defined treatment scheme in 54 of 69 (78%) patients. However, the appropriateness of clinical decisions cannot be evaluated based solely on VeriStrat results and the existence of erlotinib prescription, without accounting for timing of testing. The subsequent chart reviews provided more detailed insight into the clinical process.

**Fig. 3:**
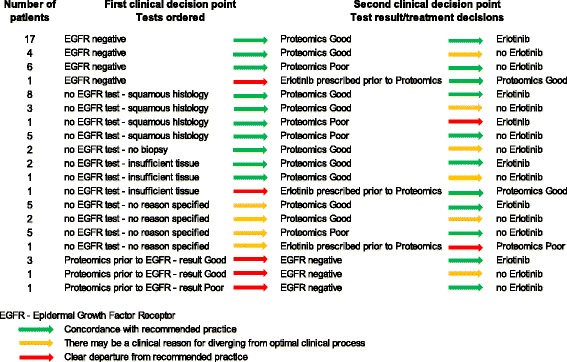
Timing of clinical decisions for EGFR and proteomic testing and use of erlotinib

Detailed chart reviews revealed the timing of clinical decisions regarding testing and treatment for each patient. These results are summarized in Fig. 3. We grouped the clinical decisions into three categories. Clinical decisions that are concordant with recommended practice are illustrated as green. Decisions that depart from recommendations but may have a valid clinical reason for the departure are coded as yellow. Decisions that are clearly not concordant with recommendations are illustrated as red.

There were 28 patients who had documented EGFR-negative status prior to ordering the proteomic test. Of those, 27 cases were coded with green arrows with respect to the first clinical decision, because the purpose of proteomic testing is to identify those patients who might respond to erlotinib. One patient had erlotinib prescription prior to proteomic testing. This case was coded as red because if the proteomic test is used, the results should be obtained prior to erlotinib treatment. Among the 27 patients, 21 had proteomic “Good” status and 17 of those received erlotinib following testing, while 4 did not receive the drug. The 4 patients who did not receive erlotinib were illustrated in yellow because the proteomic test appears to have been ordered for prognostic information only rather than to inform the decision to prescribe erlotinib. The remaining 6 patients who were EGFR negative and proteomics “Poor” are coded in green because they did not receive erlotinib.

There were 36 patients who did not have a record of EGFR testing. Six of those patients either had no biopsy or insufficient amount of tissue was available for testing. These patients were eligible for proteomic testing to determine erlotinib benefit. For the remaining 30 patients there was no reason for lack of EGFR testing documented in the clinical notes. Squamous histology explains lack of EGFR testing in 17 patients, so these decisions were coded as green. Clinical guidelines recommend that all patients diagnosed with advanced, non-squamous NSCLC be tested for EGFR. However, there may have been insufficient biopsy tissue to conduct the EGFR test or the patient may have been too weak to undergo a biopsy. Because we were unable to determine the reason for no EGFR test in 13 patients with non-squamous histology, we coded these decisions as yellow.

For 25 of the 36 patients, the decision regarding erlotinib administration was in agreement with the proteomic results: 15 patients were proteomic “Good” and received erlotinib, while 10 were proteomic “Poor” and did not receive the drug. Both clinical decisions were coded as green because these were concordant with guidelines. However, 8 patients with proteomic “Good” status did not receive erlotinib. We coded proteomic good results with no erlotinib treatment as yellow because the test is apparently being used for prognostic purposes rather than to inform treatment decisions. One patient received erlotinib despite “Poor” proteomic status, and 2 patients had erlotinib prescription prior to the proteomic test. These decisions were coded as red.

The remaining 5 patients in our cohort received the proteomic test prior to EGFR testing, which only later revealed EGFR mutation-negative status for each patient. Although the erlotinib decision was in agreement with the proteomic result in 4 out of 5 cases, the timing of testing and treatment was a clear departure from recommended practice. The fifth patient did not receive erlotinib despite “Good” proteomic status, so in this case the timing of testing was coded as red and the treatment decision as yellow.

To summarize, among our cohort of 69 patients, we analyzed the total of 138 clinical decisions surrounding EGFR testing, proteomic testing, and erlotinib treatment. The majority (105, or 76%) of these clinical decisions were concordant with the intended use of the test. However, there were 24 (17%) decisions in which documentation of the reason for departure from clinical practice guidelines, or the timing of EGFR/proteomic test order or erlotinib treatment could have been more concordant with guidelines. Only 9 (7%) clinical decisions represented clear departures from recommendations.

Chart reviews also revealed that 9 patients underwent proteomic testing within 3 months of their death. Three of these patients were also prescribed erlotinib within this time period and two of these had proteomic “Poor” status.

Table 2 provides examples of four clinical scenarios that clearly departed from recommendations. In 3 of the 4 scenarios, erlotinib was prescribed without documentation of an EGFR mutation and without information about proteomic status. Most of these clinical decisions can be characterized as compassionate use of erlotinib. Yet, these were costly decisions that provided no benefit to the patient and may have impeded use of standard, platinum based chemotherapy. In the first scenario, the patient suffered negative side effects of erlotinib, which could have been prevented if the oncologist had initially ordered an EGFR test or a proteomic test. In the second scenario, the patient continued taking erlotinib even after getting the proteomic “Poor” result. In the third scenario, the erlotinib prescription and the proteomic test result were ordered at the same time. However, it took six weeks for the test to be approved, sent to the laboratory, and results to be returned. In the fourth scenario, the EGFR test was ordered after the proteomic test. EGFR testing should have been ordered first.

**Table 2:** Description of some case scenarios in which timing of testing and treatment departed from recommendations

Diagnosis	Clinical scenario
Diagnosed with squamous cell carcinoma in 2012	EGFR test was not performed. Erlotinib (100 mg) was prescribed in 2014. Three weeks later, erlotinib was put on hold due to side effects (skin rash and itching), and restarted after another month. Proteomic testing was done 5 weeks later, and the result of test was “Poor”. The physician made the decision to discontinue erlotinib. The patient received erlotinib for the total of 63 days (prescription for 3 months’ supply).
Diagnosed with adenocarcinoma in 2010	EGFR test was not performed. Erlotinib (150 mg) was prescribed for the patient in 2013. The proteomic test was done two weeks after the erlotinib prescription. The result of the proteomic test was “Poor”. The patient continued to take Erlotinib. The patient received erlotinib for 28 days (1 month’s supply).
Diagnosed with squamous cell carcinoma in 2013	EGFR test was performed at diagnosis. Result was negative. Patient's lung cancer progressed. Erlotinib was prescribed in 2014. At the same time, the proteomic test was ordered. Proteomic test results were received six weeks after erlotinib prescription. The test result was Good.
Diagnosed outside the VA, with metastatic adenocarcinoma	No EGFR test results available. Physician ordered VeriStrat in 2015. The result was “Poor”. Physician conducted a biopsy and ordered EGFR test 2 months after proteomic test results were reported. EGFR test was negative. Erlotinib was never prescribed.
Relevant costs	
EGFR test: $500	
Proteomic test: $2,112	
Erlotinib, 100 mg tablets, 1 month’s supply: $4,815	

Analysis of VA administrative data as of August 2015

## Discussion

This study illustrates the significant complexity imposed by the use of precision medicine tools in clinical care of NSCLC patients. There are approximately 7600 veterans diagnosed with lung cancer each year. Over the period studied (18 months), based on histologic type (non-squamous NSCLC) and stage (IV), approximately 2280 patients were eligible for EGFR mutational testing. Only 69 patients (3%) underwent proteomic testing, which illustrates how rare this testing is within the VA. Yet, there are potentially many more patients who could benefit from proteomic testing. Our prior study of EGFR testing in the VA found that 64 (7%) out of 973 patients tested had sensitizing mutations [13]. Theoretically, all the EGFR-negative patients or those lacking tumor tissue are eligible for VeriStrat if the oncologist is considering prescribing erlotinib. However, it is extremely difficult, if not impossible, to determine the number of patients whom the oncologist considers a good candidate for erlotinib or those who do not have tissue available.

We found that the majority of clinical decisions surrounding the use of proteomic testing were concordant with the test’s intended use. Yet, EGFR testing, the least expensive clinical intervention analyzed in this study, was significantly underutilized within our cohort. These data are in agreement with our recent research on EGFR testing at 70 VA medical centers between 2011 and 2013. In that study, we found that only 22% of lung cancer patients eligible for EGFR testing (based on their histologic classification and stage) received the test [13]. The high rate of smoking among veterans may be one of the reasons for underutilization of EGFR testing at the VA. In the current study, more than half of the patients who received proteomic testing lacked information on EGFR mutation status. Proteomic testing costs 4 times that of EGFR testing. However, because it is a simple blood test, it is much easier for clinicians to order and easier for patients to provide a sample.

As the number of precision medicine tools expands, there is the risk that clinicians and patients choose the easiest testing and treatment decisions rather than the algorithm that has the strongest evidence of clinical and cost effectiveness. Our analysis provided several clinical scenarios illustrating that the timing of proteomic testing and erlotinib diverged from the recommended algorithm. These examples resulted in excessive costs of care, with no documented improvements in health outcomes. In one case, the patient suffered a negative response to erlotinib.

Appropriate use of genomic and proteomic testing can improve health outcomes and decrease healthcare costs associated with ineffective interventions. However, there are relatively few studies analyzing real-world use of testing and targeted treatments in routine clinical care. Precision medicine has greatly increased the complexity of care yet most EHRs, including the VA’s, do not accurately capture biomarker test orders and results or link these results to pharmacy databases to inform prescribing practices. The VA’s laboratory informatics management system lacks the capacity to capture and store orders and results from precision medicine tests. Test orders and results are either captured as unsearchable image files, entered as free text in clinical notes, or are not recorded in the EHR. Decision support systems to guide testing and treatment decisions depend on improving the laboratory information management within EHRs. These limitations make it very difficult for healthcare systems to study clinical utility, health outcomes, and cost effectiveness. The VA has recently undertaken several initiatives to improve healthcare coordination and clinical decision support systems. Results of precision medicine tests are going to be captured directly from laboratories as structured data and incorporated into the EHR. Further, VA’s Connected Health division has developed a mobile application that provides veterans and VA care teams with laboratory test results. VA is also developing applications that link results of precision medicine test with pharmacy data so that treatments are more reliably informed by test results. These improvements will facilitate appropriate use of precision medicine within the VA health system.

## Conclusions

Our analysis of electronic health records of lung cancer patients who received proteomic testing at the VA revealed that EGFR testing was significantly underutilized or not properly documented. Proteomic testing was mostly concordant with recommendations. However, we identified several clinical scenarios where the timing of proteomic testing and erlotinib administration diverged from the recommended algorithm, resulting in excessive costs of care with no documented improvements in health outcomes. Clinical decision support systems integrated with EHRs would improve patient care.

## Abbreviations

CDW: Corporate data warehouse
EGFR: Epidermal growth factor
EHR: Electronic health record
FDA: Food and drug administration
NSCLC: Non-small-cell lung cancer
TKI: Tyrosine kinase inhibitor
VA: Department of Veterans Affairs
VISN: Veterans integrated service network
